# Triterpenoids Display Single Agent Anti-tumor Activity in a Transgenic Mouse Model of Chronic Lymphocytic Leukemia and Small B Cell Lymphoma

**DOI:** 10.1371/journal.pone.0000559

**Published:** 2007-06-27

**Authors:** Christina L. Kress, Marina Konopleva, Vanesa Martínez-García, Maryla Krajewska, Sophie Lefebvre, Marc L. Hyer, Teresa McQueen, Michael Andreeff, John C. Reed, Juan M. Zapata

**Affiliations:** 1 Burnham Institute for Medical Research, La Jolla, California, United States of America; 2 Department of Blood and Marrow Transplantation, University of Texas M.D. Anderson Cancer Center, Houston, Texas, United States of America; 3 Centro de Biología Molecular Severo Ochoa, Universidad Autonóma de Madrid, Madrid, Spain; The Scripps Research Institute, United States of America

## Abstract

**Background:**

The synthetic triterpenoid 2-Cyano-3,12-Dioxooleana-1,9-Dien-28-Oic Acid (CDDO) and derivatives display anti-tumor activity against a variety of cultured tumor cell lines and in mouse xenografts. In this report, we have studied the effects of CDDO and its imidazolide derivative (CDDO-Im) on chronic lymphocytic leukemia (CLL), using patients' CLL cells and a mouse model of CLL and small B cell lymphoma (SBL).

**Principal Findings:**

CDDO and CDDO-Im efficiently induced apoptosis of malignant human and mouse B-cells *ex vivo*, although CDDO-Im was over 10-fold more potent than CDDO. Treating mice with CLL/SBL with liposome-formulated CDDO or CDDO-Im resulted in significant reductions of B cells in blood, spleen and lung. CDDO-Im was shown to be more potent than CDDO, while treatment with empty liposomes had no impact on disease. CDDO-Im treatment initially resulted in an increase of circulating B cells, which correlates with a reduction in resident lymphocytes in spleen, and lungs, suggesting that CDDO-Im induces mobilization of tumor cells from lymphoid organs and infiltrated tissues into the circulation. Analysis of blood cells recovered from treated mice also showed that CDDO-Im is a potent inducer of tumor cells death *in vivo*. Furthermore, CDDO-Im efficiently eradicated mouse CLL/SBL cells but had little effect on the viability of normal B and T cells *in vivo*.

**Significance:**

The presented data demonstrate that triterpenoids CDDO and CDDO-Im reduce leukemia and lymphoma burden *in vivo* in a transgenic mouse model of CLL/SBL, and support the clinical testing of CDDO-based synthetic triterpenoids in patients with CLL.

## Introduction

Chronic Lymphocytic Leukemia (CLL) is the most common leukemia in the western world, and it is characterized by the gradual accumulation of quiescent, apoptosis resistant, B cells [1], [2]. No cure is available at this time for this disease, although the current therapies involving the use of chemotherapeutical drugs, such as the purine analog F-ara-A, have proven helpful for some patients. However, a significant number of patients will eventually develop refractory disease as a result of the emergence of leukemic clones resistant to drugs, leading to patient demise [3], [4]. Consequently, new therapies are necessary for treating patients with refractory disease. However, the absence of cell lines derived from CLL patients that can propagate in mice, and the lack of mouse models of CLL that recapitulate the human disease have hampered the preclinical development of new therapies and treatment strategies for this disease.

Overexpression of both Bcl-2 and TRAF1 is a hallmark of human CLL cells [5], [6]. Bcl-2 overexpression largely accounts for the apoptosis resistant phenotype of CLL cells [5]. TRAF1 is frequently overexpressed in CLL cells from patients with refractory disease, suggesting that TRAF1 might be involved in disease progression [7]. Indeed, mice overexpressing in B cells both a TRAF2 mutant that mimics TRAF1 and Bcl-2 develop Small B-cell Lymphoma (SBL) and Chronic Lymphocytic Leukemia (CLL) with high incidence [8]. This TRAF2 mutant consists of only the TRAF domain and thus, like TRAF1, is devoid of the RING and zinc finger domains found in other members of the TRAF family [9]. Furthermore, CLL/SBL cells from the TRAF2DN/Bcl-2 mice are resistant to chemotherapeutic drugs, such as dexamethasone and F-ara-A [8]. Altogether, the CLL developed by these mice recapitulates many aspects of refractory CLL disease in humans, and as such, this CLL mouse model might be suitable as preclinical model for testing new drugs for CLL.

Triterpenoids, such as oleanolic acid and ursolic acid, are natural compounds with anti-tumorigenic and anti-inflammatory properties [10]. Synthetic triterpenoid derivatives such as 2-Cyano-3,13 dioxooleana-1,9(11)-dien-28-oic acid (CDDO) [11] and its derivative 1-[2-cyano -3-,12-dioxooleana-1,9(11)-dien-28-oyl]imidazole (CDDO-Im) [12] have more potent anti-tumoral activity compared to the natural compounds. Thus, they efficiently inhibit tumor cell proliferation [13]–[18] and induce apoptosis of a variety of epithelial tumor cell lines [13], [16], [19]–[22] , lymphoid cell lines and patient-derived leukemia specimens [17], [23]–[27]. Interestingly, CDDO and CDDO-Im have been shown to efficiently induce apoptosis of multiple myeloma cells that were resistant to conventional therapies with little effect on the viability of normal cells [26], [28]. Triterpenoids also restore the sensitivity of resistant tumor cells to TRAIL [19], [21], TNF [27], [29] and chemotherapeutic drugs [26], and are well tolerated by mice [13], [14], [18], [21].

The current evidence suggests that synthetic triterpenoids have a variety of effects on apoptosis pathways, which might be different depending on the cell-type under investigation. In this regard, triterpenoids have been shown to induce depletion of mitochondrial glutathione [22], [28], [30], to inhibit mitochondria electron transport [22], [23], [26], [28], [31], to increase the concentration of reactive oxygen species [22], [28], to inhibit NFκB activation [27], [29], [32], to downregulate FLIP [19], [21], [28], [33] and upregulate TNF-family Death Receptors [20], [21], [30], resulting in cell death by activation of caspase-dependent [20], [23]–[26], [28], [29], [33], [34] and independent [24] cell death pathways.

In this report, we used TRAF2DN/Bcl-2 mice that have developed CLL/SBL as a preclinical model to test the efficacy of CDDO and CDDO-Im against CLL cells, both *ex vivo* and *in vivo*.

## Materials and Methods

### Transgenic mice

Transgenic mice expressing both Bcl-2 and a TRAF2 deletion mutant lacking the N-terminal 240 amino-acids encompassing the RING and zinc finger domains (TRAF2DN) have been described [8]. Bcl-2 (Balb/c) and TRAF2DN (FVB/n) heterozygous mice were bred to produce TRAF2DN/Bcl-2 double-positive mice. Mouse genotyping was performed by PCR analysis using primers specific for human Bcl-2 and TRAF2DN.

### Cell isolation

Mouse spleens were carefully crushed to release the lymphocytes. Blood was collected from the cavernous sinus and collected in tubes coated with heparin. The animal protocols were approved by the Institutional Animal Care and Use Committee. Euthanasia was performed by following the rules of the American Veterinarian Medical Association. Patient CLL cells were obtained from the Chronic Lymphocytic Leukemia Research Consortium (CRC) (Moores Cancer Center, University of California San Diego, La Jolla, CA). Donations of CLL samples to CRC require the written consent of the patients in compliance with the Declaration of Helsinki.

### Liposome production

CDDO and CDDO-Im were formulated in liposomes at a concentration of 2 mg/ml as described [18]. Briefly, triterpenoids (CDDO and CDDO-Im) were solubilized in t-butanol at 37°C at a concentration of 2 mg/ml. Phospholipid distearoyl phosphatidyl choline (DSPC) was solubilized in t-butanol at 55°C, at a concentration of 10 mg/ml. DSPC and either CDDO or CDDO-Im were then mixed together and frozen. The lipid/drug ratio used was 20∶1. Liposomes containing triterpenoids were lyophilized overnight and then reconstituted in normal saline at 55°C, and centrifuged at 13,000 rpm for 1 hour. Pellets were resuspended at room temperature in normal saline at a concentration of 2 mg/ml (100 µM) for the *in vivo* studies. Empty liposomes were made using the same lipids and following the same protocol, but without adding triterpenoids.

### Drug administration

TRAF2DN/Bcl-2 mice were injected into the tail vein with empty liposomes or liposomes containing either CDDO or CDDO-Im, at 5, 10 or 20 mg/kg per dosage (corresponding to 17.3, 34.5 and 69 mg/m^2^/dosage, respectively). Each mouse received nine injections administered over a period of 21–25 days. The concentration of B cells in blood was monitored every other day after inoculations, and 3 to 10 days after the final inoculation.

### Quantification of B and T cells in blood

Blood was collected in heparinized capillary tubes and diluted 5 times in PBS containing 10% FCS, 1% BSA, 0.05% sodium azide, 5 mM EDTA and 50 µg/ml of human γ-globulin and incubated for 15 min at RT. Then, the mixture was incubated with anti-CD45 PE-Cy5 and either anti-B220 PE, anti-CD4 PE or the respective isotype controls, and incubated for 20 min at RT. Erythrocytes were lysed using 10 volumes of hypotonic lysis buffer (PharMlyse, BD biosciences) and quantification was performed on a personal cell analysis and counter-top microfluorocytometer (Guava technologies, Hayward, CA).

### Flow cytometry

Cell suspensions were depleted of red cells and neutrophils by density centrifugation (Lympholite-M, Cedarlane laboratories, Hornby, Ontario). Lymphocytes were incubated with 50 µg/ml human γ-globulin to block Fc-receptors. Then 10^5^ to 5×10^6^ cells were incubated with a combination of allophycocyanin (APC)-, fluorescein isothiocyanate (FITC)- or phycoerythrin (PE)-conjugated antibodies recognizing various surface markers. After 1h incubation at 4°C, cells were washed in high glucose DMEM (without phenol red) (Irvine Scientific, Santa Ana, CA) containing 3% FCS (Hyclone, Logan, UT). Flow cytometry analysis was accomplished using a FACScalibur equipped with detectors for 4 colors (BD biosciences, San Jose, CA).

### Cell viability

CLL cells from human patients were incubated *ex vivo* with or without triterpenoids. Cells were harvested after 24 h, washed, incubated with Annexin-V-FITC and propidium iodide (PI), and analyzed by flow cytometry. In the case of mouse splenocytes, cells were harvested 24 h after the addition of the drugs to cells. Then, cells were incubated with APC-anti-B220 mAb for 30 min, washed and incubated with Annexin-V-FITC and PI and then analyzed by flow cytometry.

Viability of blood leukocytes was determined as follows: blood (20 µl) was collected from the cavernous sinus and immediately mixed with 80 µl of PBS containing 10% FCS, 1% BSA, 0.05% sodium azide, and 5 mM EDTA. Then, 15 µl of this mixture was subjected to hypotonic lysis with 250 µl of PharM Lyse (BD-biosciences). After incubation for 20 minutes, cells were recovered by centrifugation for 5 minutes at 2,500 rpm, and the resulting cell pellet was resuspended in 200 µl of Guava ViaCount reagent (Guava Technologies) and incubated in the dark for 20 minutes. Fluorometry assay was performed using the Guava PCA-96 microfluorometer.

### Immunohistochemistry

Tissues and organs from transgenic mice of the various genotypes were fixed in Z-fix solution (Anatech Ltd, Hayward, CA) and embedded in paraffin. Tissue sections (5 µm) were stained with hematoxylin and eosin (H&E).

## Results

**Figure 1:**
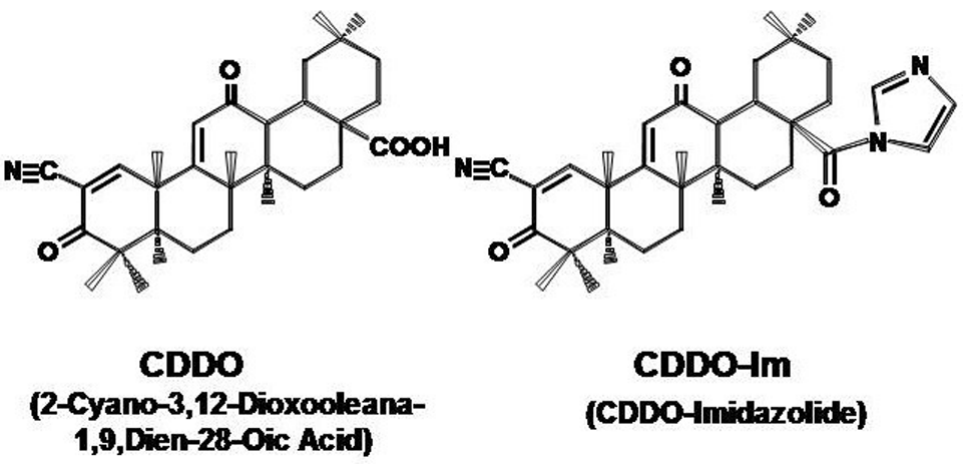
Chemical structures of CDDO and CDDO-imidazolide. The synthesis of CDDO and CDDO-Im was previously described [11], [12].

**Figure 2:**
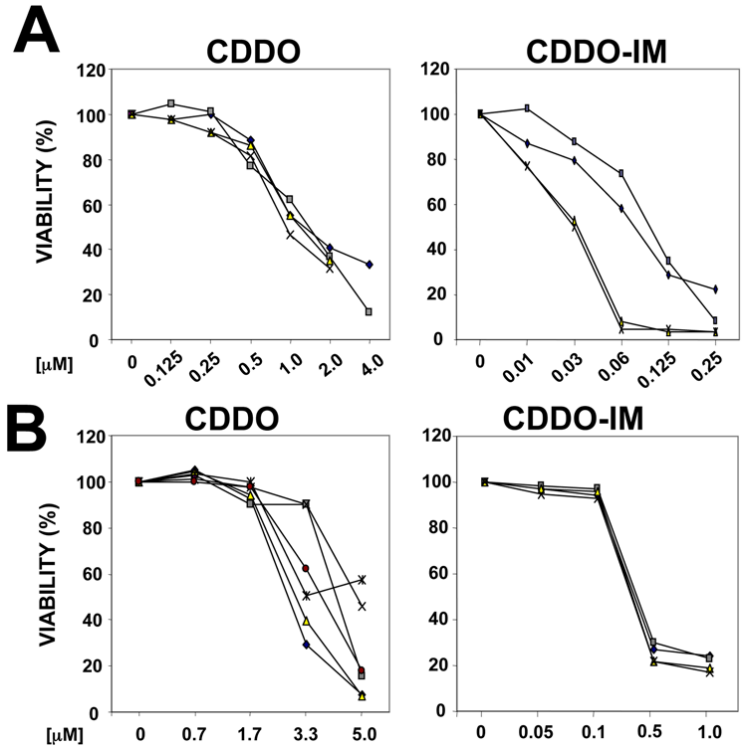
Effects of CDDO and CDDO-Im on apoptosis of human CLL and TRAF2DN/Bcl-2 cells. Human CLL cells (A) and splenocytes isolated from TRAF2DN/Bcl-2 transgenic mice (B) were cultured with or without CDDO and CDDO-Im at the indicated concentrations. Human CLL cells (n = 4) were obtained from 2 patients previously untreated with standard therapies (⋄ and X), one patient refractory to chlorambucil (▪) and one patient refractory to F-ara-A (Δ). Cells were harvested after 24 h, washed and incubated with Annexin-V-FITC and PI. Mouse splenocytes (n = 6) were also harvested after 24 h, but first they were incubated with APC-anti-B220 mAb for 30 min, then washed and incubated with Annexin-V-FITC and PI. Lymphocytes (A) or B lymphocytes (B220^+^ cells) (B) were gated and the percentage of apoptotic cells (Annexin V^+^) was determined by flow cytometry. The percentage of non-apoptotic viable cells is shown. Data were corrected for differences in spontaneous cell death.

To test whether triterpenoids CDDO and CDDO-Im (Figure 1) induce apoptosis of human and mouse CLL cells *ex vivo*, CLL cells from human patients and splenocytes from TRAF2DN/Bcl-2 double positive mice were cultured in the presence of various concentrations of CDDO and CDDO-Im. Both CDDO and CDDO-Im efficiently induced apoptosis of human (Figure 2A) and mouse (Figure 2B) CLL/SBL cells. However CDDO-Im was 10-times more active than CDDO against mouse leukemic cells, and 20-times more active against human CLL cells compared to CDDO. For mouse TRAF2DN/Bcl-2 B cells the average LD_50_ for CDDO-Im was 0.37±0.01 µM (n = 4), while it was 3.7±0.3 µM for CDDO (n = 6). In the case of human CLL cells, the average LD_50_ for CDDO-Im was 0.06±0.017 µM compared to 1.26±0.125 µM for CDDO (n = 4).

**Figure 3:**
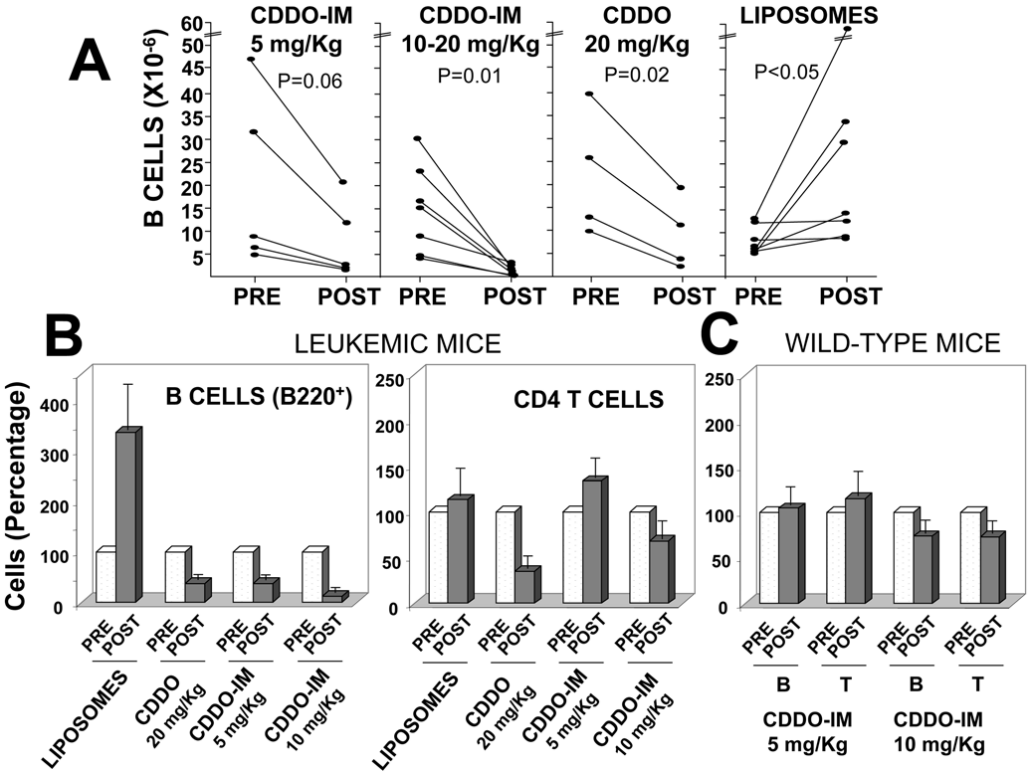
In vivo effect of CDDO and CDDO-Im on CLL/SBL cells and normal lymphocytes (A). Mice with leukemia were treated as indicated. Total numbers of circulating B cells (B220^+^) in blood were quantified 1–2 days before the beginning of the treatment (pre) and 7–10 days after the end of treatment (post). Statistical significance was determined by Student's two-tailed paired t-test. (B) Total numbers of circulating B (B220^+^) and T (CD4^+^) cells were quantified in blood before and after treatment with empty liposomes (n = 5), CDDO (20 mg/Kg; n = 5), or CDDO-Im at 5 (n = 6) or 10 mg/Kg (n = 9). The ratio of the concentration of cells before and after the treatment was calculated. Figure shows the average percentages ±SEM. (C). Wild-type mice were treated with 5 or 10 mg/Kg CDDO-Im and the concentration of B (B220+) or T (CD4+) lymphocytes in blood before and after the treatment was determined. Figure shows the average percentages ±SEM as described in B.

Next, we assessed the efficacy of CDDO and CDDO-Im *in vivo*. For these studies, we used TRAF2DN/Bcl-2 mice that have developed leukemia, as indicated by the presence of a majority of CLL (FSC^M^ B220^M^) cells in blood (over 4×10^6^ B cells/ml). Mice at this stage of the disease already have severe splenomegaly and lymphadenopathy, and the leukemia progresses over time (2–4 months) to over 50×10^6^ CLL B cells/ml [8]. Groups of TRAF2DN/Bcl-2 mice that had developed leukemia were injected i.v. with empty liposomes or with liposomes containing either CDDO (20 mg/Kg) or CDDO-Im, at doses of 5, 10 or 20 mg/kg/day (Table 1 and Figure 3A). Each mouse received a total of nine injections of a drug administered every 2–3 days over a period of 21–25 days. The concentration and viability of B cells in blood was monitored the day after each inoculation and 3–10 days after the end of the treatment. As shown in table 1, treating TRAF2DN/Bcl-2 mice with empty liposomes had no significant impact on blood B cell counts or on the normal progression of the leukemia (8.2±1.4 vs 26.2±6.8 millions B cells/ml before and after the treatment, respectively). In contrast, CDDO-treated mice (20 mg/Kg) showed a progressive reduction in the number of B cells in blood throughout the course of the treatment, with an average 60% reduction in the number of B cells in blood at the end of the treatment (22.75±7 vs 9.5±3.8 million B cells/ml before and after the treatment, respectively). In agreement with the *ex vivo* data (Fig. 2), the anti-tumor effect of CDDO-Im *in vivo* was also significantly greater than CDDO. Indeed, treatment with 5 mg/Kg CDDO-Im induced a similar reduction in the number of B cells in blood (average 60% reduction; 39.4±20 vs 16.5±9) as achieved with 20 mg/Kg CDDO. Treating mice with 10 or 20 mg/Kg CDDO-Im resulted in reductions of over 90% of B cells in blood (average 14.5±3.7 vs 1.3±0.4 millions B cells/ml before and after the treatment, respectively) (Figure 3A and B).

**Table 1:** Concentration of B cells in blood of TRAF2DN/Bcl2 mice treated with empty liposomes or with triterpenoids.

mouse ID	Drug	B cells (millions)					
	dosage	pre-treatment	1st dosage	2nd dosage	3th dosage	6th dosage	post-treatment
	Liposomes	Day -1	Day 1	Day 2-3	Day 4-6	Day 11-14	Day 24 to 28
**310**	–	7	8	9	9	6	7
**327**	–	12	nd	10	8.4	8	12
**325**	–	13	10	15	13	14	58
**420**	–	6	7	8	nd	7	13
**414**	–	5	5	5	5	7	27
**413**	–	6	8	10	6	23	34
	**CDDO**						
**209**	20 mg/Kg	26	10	22	21	18	11
**332**	″	14	8	11	11	14	5
**190**	″	10	12	6	7	4.5	2.4
**300**	″	41	26	34	45	48	19.6
	**CDDO-Im**						
**614**	5 mg/Kg	32	70	77	35	35	12
**615**	″	5	9	6.4	9	3.2	1.5
**655**	″	6.6	9.5	10.6	5.3	7.8	2.3
**691**	″	48	46	64	73	77	21.6
**694**	″	9.5	19	17	17	7.5	3.1
**711**	″	134.7	123	119	140	126	58.5
**203**	10 mg/Kg	14	63	60	71	dead	–
**224**	″	23	32	24	31	12	2
**227**	″	13	53	nd	69	dead	–
**389**	″	9	39	42	50	10	3.3
**314**	″	4	24	32	15	20	0.6
**317**	″	7	38	32	20	14	dead
**349**	″	30	50	47	37	27	0.8
**365**	″	4.3	21	25	12	5	0.5
**382**	″	7	14	29	20	dead	–
**302**	20 mg/Kg	26	240	dead	–	–	–
**343**	″	16	28	72	21	7	1.5
**344**	″	15	nd	41	15	2	0.6
**369**	″	23	140	173	118	dead	–
**388**	″	49	105	163	130	dead	–

TRAF2DN/Bcl-2 mice were injected i.v. with empty liposomes or with liposomes containing either CDDO or CDDO-Im at doses of 5, 10 or 20 mg/kg/day (corresponding to 17.3, 34.5 and 69 mg/m^2^/day, respectively). Each mouse received a total of nine injections administered over a period of 21–25 days. The concentration of B cells (×10^6^/ml) in blood was monitored one day before the inoculation with drug (pre-treatment), after the inoculation of the 1^st^, 2^nd^, 3^rd^ and 6^th^ drug dosages, and 3 to 10 days after the final inoculation(post-treatment). (nd: not determined).

CDDO and CDDO-Im treatments also reduced the number of T cells in blood (Figure 3B). However, while CDDO seems to reduce the numbers of malignant B cells and normal T cells to similar extents, CDDO-Im seems to be comparatively more toxic to mouse leukemic B cells than to normal T cells. Indeed, even at concentrations of CDDO-Im that reduced 90% of B cells in blood, T cells (CD4+) are only reduced an average of 30% (Figure 3B).

The limited toxicity of CDDO-Im against normal lymphocytes was confirmed by treating wild-type mice with 5 and 10 mg/Kg CDDO-Im, using the same inoculation schedule that was used for treating the leukemic mice. As shown in Figure 3C, treatment with 5 mg/Kg CDDO-Im had no deleterious effect on normal B and T levels in blood, while treatment with 10 mg/Kg CDDO-Im resulted in a moderate 30% reduction in both B and T lymphocytes in blood. Moreover, treatment of wild-type and leukemic mice with CDDO-Im caused a moderate weight loss which stabilizes or even started to recover by the end of the treatment (Figure S1).

Furthermore, wild-type and CLL/SBL mice treated with empty liposomes (vehicle; n = 6), CDDO (20 mg/Kg; n = 4) or with 5 mg/Kg CDDO-Im (n = 8) survived the treatment (Table S1). In contrast, while a dosage of 10 mg/Kg CDDO-Im was harmless to wild-type mice (n = 7), 44% of CLL/SBL mice (n = 9) died during treatment (Table 1 and Table S1). This result suggests that 10 mg/Kg CDDO-Im is not intrinsically toxic to mice, and that the mortality of CLL/SBL mice might be related to the effect of the drug on the leukemic cells. Finally, 33% of wild-type and 60% of the leukemic mice treated with 20 mg/Kg CDDO-Im (n = 5), died during treatment (Table 1 and Table S1).

Immunohistochemical analysis of the tissues of mice treated with CDDO-Im that died during treatment showed evidence of kidney and liver toxicity, as indicated by hemorragies, cytoplasmic eosinophilia and vacuolarization in the kidneys, and nuclear pyknosis, hepatocellular necrosis and acidophilia in the liver (not shown). These toxic reactions were significantly milder in wild-type mice or in those mice with leukemia that survived the treatment.

**Figure 4:**
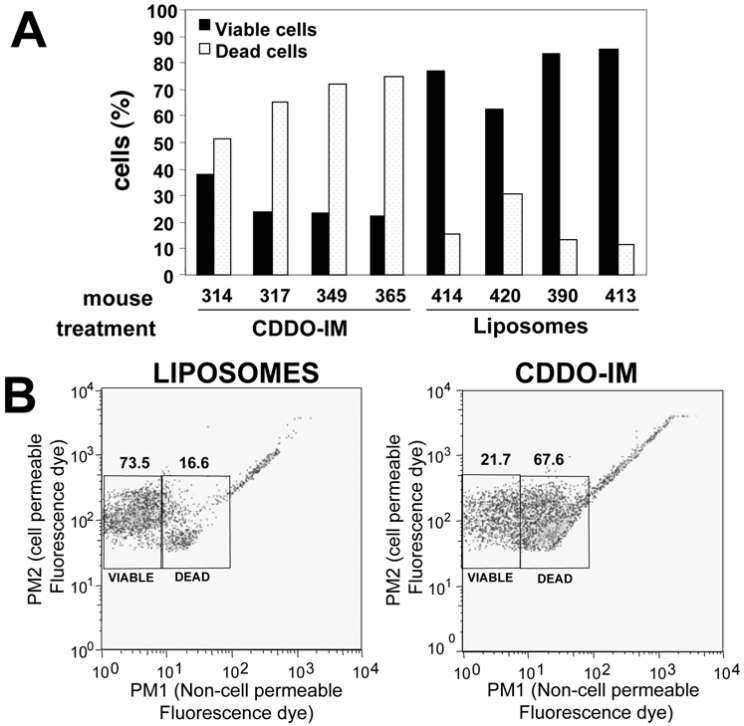
CDDO-Im induces massive cell death *in vivo*. **A.** The percentage of viable (black columns) and apoptotic (dotted columns) cells in the blood of representative mice after 3 inoculations with empty liposomes or liposomes containing CDDO-Im is shown. Viability was assessed using an assay based on the alterations in the permeability of cells at different viability stages using a combination of DNA-binding dyes (ViaCount, Guava Technologies) and a Guava PCA96 fluorometer and analyzed with Guava's multi-caspase software. **B.** Representative flow cytometry profile of the viability of blood cells from mice treated with empty liposomes or liposomes containing CDDO-Im using Viacount (Guava Technologies). Analysis was performed with Flowjo software (Tree Star, Inc, Ashland, OR). PM2 (red channel) shows nuclear staining of cells using a cell permeable dye. PM1 (yellow-orange channel) shows staining of non-viable cells with a non-cell permeable dye.

Next, we determined the effect of CDDO-Im on the viability of blood cells *in vivo*. These analyses were performed using an assay based on the alterations in the permeability of cells using a combination of DNA-binding dyes. As shown in Figure 4A, CDDO-Im induced massive blood cell death. Dead cells are already detected after the first dosage of drug, reaching a peak after the third inoculation with CDDO-Im (Figure 4A). Representative examples of cell viability data showing the effect of empty liposomes and CDDO-Im are shown in Figure 4B.

**Figure 5:**
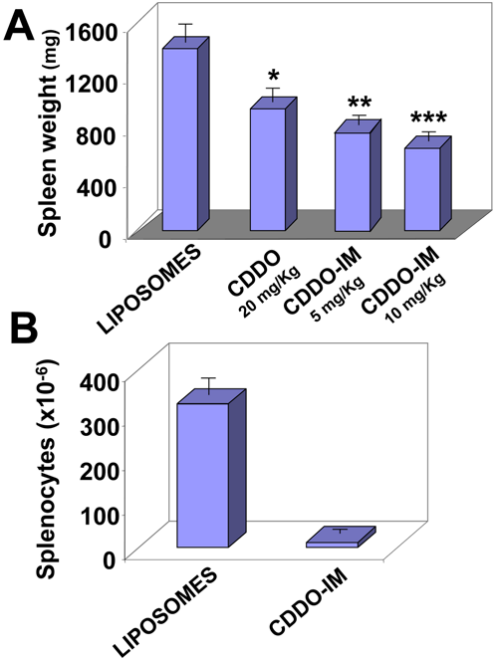
Amelioration of splenomegaly by CDDO and CDDO-Im treatment. **A.** TRAF2DN/Bcl-2 mice with leukemia were treated with empty liposomes or with liposomes containing either CDDO (20 mg/Kg/day) or CDDO-Im (5 or 10 mg/Kg/day). Mice were euthanized 7–10 days after the final drug administration, and the spleens were weighted. The average weight ±SEM of the spleens after the different treatments was: empty liposomes: 1405±133 mg, n = 7; CDDO: 937±117, n = 4; CDDO-Im (5 mg/Kg): 821±83, n = 4; and CDDO-Im (10 mg/Kg): 635±71 mg, n = 6. Statistical significance of liposomes vs CDDO (*, p = 0.04) and liposomes vs CDDO-Im (5 mg/Kg**, p = 0.013; 10 mg/Kg*** p = 0.0005) was determined using unpaired t-test. **B.** Total number of lymphocytes isolated from spleens of mice treated with empty liposomes (324±44.7×10^6^; n = 3) or with liposomes containing CDDO-Im (10 mg/Kg) (12.7±6.7×10^6^; n = 3) is shown.

Those mice that completed the treatment were euthanized 7 to 10 days after the final dosage of drug and tissues were analyzed. Mice treated with empty liposomes had severe splenomegaly (1405±133 mg, n = 7) (Figure 5A) and lymphadenopathy, similar to untreated TRAF2DN/Bcl-2 mice with overt disease [8]. In contrast, mice treated with CDDO (20 mg/Kg) showed a significant reduction in the spleen weight (937±117 mg; n = 4), which was similar to the reduction achieved with 5 mg/Kg CDDO-Im (839±83 mg; n = 4). This reduction in spleen weight was even more striking for mice treated with 10 mg/Kg CDDO-Im (635±71 mg; n = 6) (Figure 5A). The spleens of CDDO-Im-treated mice contained less than 5% the total number of splenocytes compared to spleens of mice treated with empty liposomes (Figure 5B). In addition, CDDO-Im-treated mice showed little or no residual lymphadenopathy (not shown).

**Figure 6:**
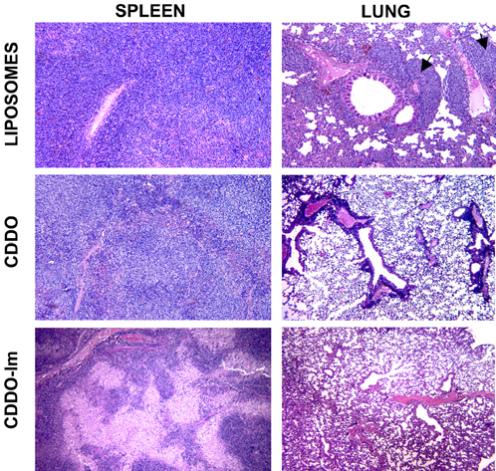
Histochemical analysis of the spleen and lung of leukemic mice treated with empty liposomes or liposomes containing CDDO or CDDO-Im. Tissues and organs from TRAF2DN/Bcl2 leukemic mice were treated as indicated in the figure. After treatment, tissues were fixed in Z-fix solution (Anatech Ltd.), embedded in paraffin, and tissue sections (5 µm) were stained with hematoxylin and eosin (H&E). One representative example for each treatment is shown. Arrows indicate the presence of infiltrating lymphocytes.

TRAF2DN/Bcl-2 mice with overt disease have an altered splenic architecture caused by the accumulation of leukemic B cells and they also have lymphocyte infiltration into several organs. In particular, mice at the final stages of disease have severe lung infiltration with development of pleural effusion that seems to be the ultimate cause of death [8]. Histochemical analysis of spleens and lungs of TRAF2DN/Bcl-2 mice treated with empty liposomes demonstrated massive accumulation of CLL/SBL cells in these organs (Figure 6), similar to that observed in untreated mice with overt disease. Treatment with CDDO reduced the density of lymphocytes in spleen and significantly diminished lymphocyte infiltration in lungs. However, the most striking improvement was observed in mice treated with CDDO-Im. Histochemical analysis of spleens of TRAF2DN/Bcl-2 mice with overt disease following treatment with CDDO-Im showed a dramatic reduction in resident lymphocytes in spleen, and the lungs of CDDO-Im-treated mice showed essentially no lymphocyte infiltration by the end of the treatment (Figure 6).

Remarkably, mice treated with CDDO-Im showed a striking increase in circulating B cells (B220^+^) after the first three CDDO-Im inoculations, followed by a sustained reduction as the treatment progressed (Table 1). This effect was more evident in mice treated with 10 or 20 mg/Kg, but it was also seen in mice treated with 5 mg/Kg CDDO-Im. In contrast, this increase in circulating B cells was not observed in mice treated with CDDO (20 mg/Kg) (Table 1). Altogether, the striking reduction in resident lymphocytes in spleen, and lungs, and the increase in circulating leukemic cells in mice treated with CDDO-Im, suggest that this CDDO-derivative induces mobilization of tumor cells from lymphoid organs and infiltrated tissues into the circulation.

**Figure 7:**
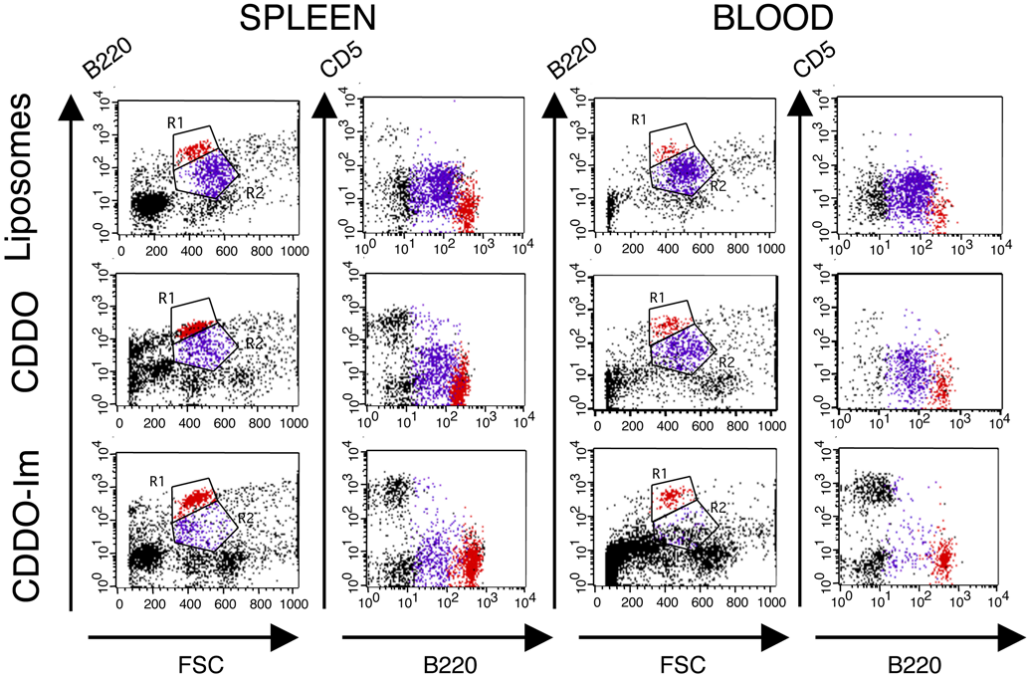
Analysis of B cell populations in spleen and blood of leukemic mice treated with empty liposomes or liposomes containing CDDO (20 mg/Kg) or CDDO-Im (10 mg/Kg). Three-color flow-cytometry analysis was performed to determine the phenotype of B lymphocytes. Two different gates were used to identify normal B cells and CLL/SBL. First, lymphocytes were selected by gating the lymphocyte population in a forward scattered (FSC) and side scattered (SSC) plot (not shown). Then, B cell populations were identified by plotting B220 expression and FCS. Gate R1 contains cells with high expression of B220 (B220^H^) and small size (FSC^L^). Red dots within gate R1 were also contained in the lymphocyte gate and represent normal B cells. Gate R2 includes cells with medium expression of B220 (B220^M^) and larger in size (FSC^M^). Purple dots within gate R2 were also contained in the lymphocyte gate and represent CLL/SBL cells. The analysis of the lymphocyte populations expressing B220 and CD5 is also shown. Representative results are provided for mice that completed the treatment with each drug.

**Figure 8:**
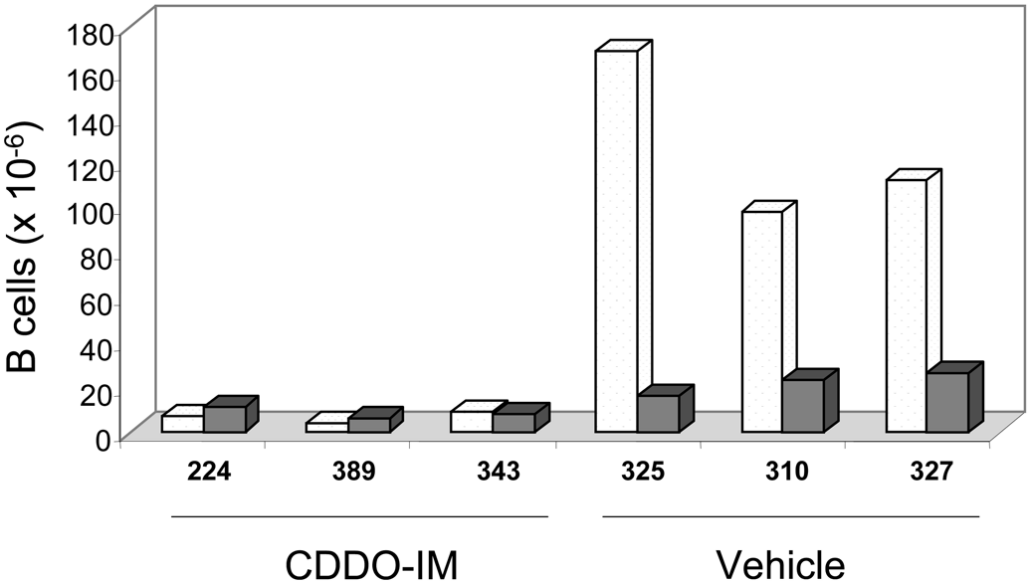
Determination of the total number of normal B lymphocytes and CLL/SBL cells in spleen of mice treated with empty liposomes or with CDDO-Im containing liposomes. The total number of B lymphocytes (CD45^+^/B220^+^) in spleen was quantified using a cell analysis and microfluorocytometer (Guava technologies). The percentages of the B220^H^ FSC^L^ normal B cells and of the B220^M^ FSC^M^ CLL/SBL cells were assessed by FACS (see Figure 6). The total number of CLL/SBL cells (white) and normal B lymphocytes (grey) in 3 mice treated with 10 mg/Kg CDDO-Im and 3 mice treated with empty liposomes is shown.

Finally, FACS analysis of the B cell populations in blood and spleen of untreated mice and of mice treated with empty liposomes showed that the majority of cells were CLL/SBL B cells. These cells are characterized by their medium size and surface expression of B220^M^ and CD5^+^, which differ from normal B cells that are small size, have surface B220^H^ and lack CD5 expression (Figure 7). Treatment with CDDO resulted in a modest reduction of CLL/SBL B cells in spleen and blood. However, the most striking reduction in leukemic B cells in blood and spleen was achieved with CDDO-Im (Figure 7). Furthermore, and confirming the results described above (Figure 3), normal B cells were much less affected by both CDDO and CDDO-Im, remaining readily detectable in blood and spleen of mice treated with CDDO and CDDO-Im (Figures 7 and 8).

## Discussion

CLL represents the most common type of leukemia. Therefore, the development of new therapies for CLL patients with refractory disease is a priority in medical oncology. Progress however has been hampered by the difficulties in producing human CLL xenografts and the absence of animal models that accurately recapitulate human CLL and that are suitable to be used as a platform for preclinical testing of new therapies.

We have previously described the generation of a transgenic mouse model of CLL/SBL. These transgenic mice were engineered to over-express in B lymphocytes a TRAF2 mutant that mimics TRAF1 and the anti-apoptotic protein Bcl-2. Both Bcl-2 and TRAF1 are commonly overexpressed in human CLL B-cells [7]. These mice develop a disease with remarkable similarities to human CLL. Furthermore, these mouse CLL/SBL cells are resistant to apoptosis induced by F-ara-A and dexamethasone. F-ara-A is used in the treatment of CLL, but patients will eventually relapse by the selection and expansion of CLL clones refractory to F-ara-A. Therefore, this mouse model of CLL/SBL recapitulates some critical aspects of refractory human CLL disease and might be used as a preclinical model to test the efficacy of new chemotherapeutic drugs against refractory CLL cells.

In this report, we tested the efficacy of CDDO and its derivative CDDO-Im both *ex vivo* and *in vivo* using human CLL cells and a transgenic CLL/SBL mouse model. Triterpenoid-derivatives have shown promise as anti-cancer agents, inducing apoptosis or inhibiting proliferation of cultured breast [18], [21], prostate [19], lung [19], [20], pancreatic [22], ovarian [15], [19], colon [16], [19], melanoma [14], leukemia [14], [23]–[25], [27], [33], [34] and myeloma [17], [26], [28], [35] cell lines. CDDO and derivatives also reduced tumor burden *in vivo* in mice bearing human tumor xenografts, without significant toxicity to normal tissues [13], [14], [18], [21].

Our studies showed that CDDO and its imidazolide derivative (CDDO-Im) are also potent inducers of apoptosis of human and mouse CLL cells *ex vivo*. Moreover, in agreement with previous reports using other cell types [14], [28], CDDO-Im is significantly more potent than CDDO.

Treating TRAF2DN/Bcl-2 mice with overt disease with liposomes containing either CDDO or CDDO-Im showed that both triterpenoids were capable of reducing tumor burden in these mice. Similar to the results obtained *ex vivo*, CDDO-Im was also more potent than CDDO *in vivo*. CDDO treated mice showed a significant improvement at the end of the treatment, with amelioration of leukemia, lymphoma, and reduced tumor lymphocyte infiltration of lungs. However, CDDO-Im had a far more striking effect in reducing tumor B cells in blood and spleen, which were reduced to almost normal levels by the end of the treatment. Indeed, the average spleen weight of TRAF2DN/Bcl-2 mice with overt disease at the end of the treatment with CDDO-Im was 635±71 mg, which closely compares to the spleen weight of TRAF2DN/Bcl-2 mice that have not yet progressed to the aggressive leukemic phase of the disease (647±54 mg [8]). Moreover, CDDO-Im-treated mice showed very mild lymphadenopathy or lymphocyte infiltration into lungs. Altogether, these observations indicate that CDDO-Im has more potent single agent activity against CLL/SBL, compared to CDDO, both *ex vivo* and *in vivo*.

The long-term benefits of the treatment of CLL/SBL mice with triterpenoids have been difficult to determine, in part due to the asynchronous onset of the disease. However, preliminary data with a small cohort of CLL/SBL mice (n = 4) treated with CDDO-Im indicate that 50% (2/4) of the mice were free of disease for over 8 months after the treatment and were subsequently euthanized. In contrast, the other two mice relapsed within 4 months after treatment. Whether additional cycles of treatment might improve this outcome and increase the life expectancy of the leukemic mice remains to be determined.

Treatment of leukemic mice with CDDO-Im initially caused an increase in circulating B cells. This phenomenon was not observed in leukemic mice treated with CDDO. The disparity between CDDO and CDDO-Im might reflect the existence of specific activities for each of these triterpenoids. In this regard, while treatment for several days with 5 mg/Kg CDDO-Im or 20 mg/Kg CDDO had similar effects in reducing circulating B cells, the initial increase in circulating B cells was only observed in CDDO-Im-treated mice. The most likely source of these new circulating lymphocytes is spleen, lymph nodes, and tissue-infiltrating lymphocytes, thus accounting for the striking reduction of lymphocytes in those compartments. The mechanism involved in the mobilization of lymphocytes from secondary lymphoid organs and tissues is not known, but it might be related to the potent anti-inflammatory activity of CDDO-Im and other triterpenoids [14], [36], [37]. This phenomenon might also have an important impact on the anti-tumor activity of CDDO-Im *in vivo*, by removing tumor cells from protective microenvironments and making them more accessible to conventional chemotherapeutic drugs. Remarkably, CDDO-Im showed stronger toxicity against leukemic B cells than against normal B or T cells. This selective anti-tumoral activity of CDDO-Im has also been observed in other studies [21], [26], [28], and further encourages the application of triterpenoids in the clinic

Although the most striking anti-leukemic effect *in vivo* was achieved with 10 mg/Kg CDDO-Im, approximately 40% of CLL/SBL mice receiving this treatment died. In contrast, wild-type mice treated with the same dosage of CDDO-Im survived, suggesting that CDDO-Im-induced mortality is not the result of a general toxic effect of the drug. We speculate that the massive induction of leukemic cell death by CDDO-Im might lead to severe kidneys and liver toxicity. However, we cannot rule out other causes of mortality, for instance a severe hemodynamic alteration caused by the sudden increase in circulating tumor cells induced by CDDO-Im. Optimizing the dosage of CDDO-Im and/or the administration schedule might help in reducing lethality while preserving the efficacy of the drug against CLL/SBL. Indeed, our data suggest that maximal anti-leukemic activity without associated mortality could be achieved at doses of CDDO-Im between 5 and 10 mg/Kg. In this regard, it is worth noting that Phase I clinical trials currently under way show that CDDO-methyl-ester (CDDO-Me), another CDDO-derivative, is tolerated without any significant toxicities in stage IV cancer patients at doses up to 600 mg/day (352 mg/m^2^/day) (Colin Meyer, Reata Pharmaceuticals, personal communication).

Altogether, the results presented above show that triterpenoids significantly reduce leukemia cells in blood, spleen and lymph nodes as well as the amount of infiltrating tumor cells in tissues and organs in a transgenic mouse model of CLL/SBL, thus illustrating the potential of triterpenoids as novel single agents for the treatment of CLL.

## Supporting Information

Figure S1 Effect of CDDO-Im on the weight of the mice Wild-type and TRAF2DN/Bcl-2 mice that have developed CLL/SBL were treated with a dosage/day of 5 or 10 mg/Kg CDDO-Im. Mice were injected 9 times over a period of 21 days. Weights were measured before each inoculation and 2 days after the final dosage (day 23).
(0.06 MB DOC)

Table S1 Lethality associated to the treatment with triterpenoids Wild-type and TRAF2DN/Bcl-2 mice that had developed CLL/SBL were treated with CDDO-Im at the indicated dosages. Each mouse received a total of nine injections administered over a period of 21–25 days.
(0.03 MB DOC)
