# Supplementary figures and images for: Triterpenoids Display Single Agent Anti-tumor Activity in a Transgenic Mouse Model of Chronic Lymphocytic Leukemia and Small B Cell Lymphoma
Source: PLoS One. 2007 Jun 27;2(6):e559. doi: 10.1371/journal.pone.0000559 (PMC1891436; doi:10.1371/journal.pone.0000559)

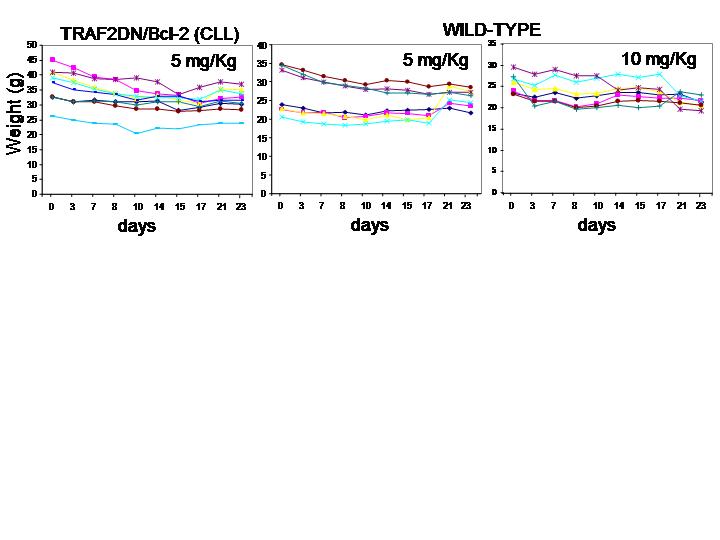

Supplement: Figure S1 — Effect of CDDO-Im on the weight of the mice Wild-type and TRAF2DN/Bcl-2 mice that have developed CLL/SBL were treated with a dosage/day of 5 or 10 mg/Kg CDDO-Im. Mice were injected 9 times over a period of 21 days. Weights were measured before each inoculation and 2 days after the final dosage (day 23). (0.06 MB DOC) [file pone.0000559.s001.doc]
