# Supplementary material for: Triterpenoids Display Single Agent Anti-tumor Activity in a Transgenic Mouse Model of Chronic Lymphocytic Leukemia and Small B Cell Lymphoma
Source: PLoS One. 2007 Jun 27;2(6):e559. doi: 10.1371/journal.pone.0000559 (PMC1891436; doi:10.1371/journal.pone.0000559)
Supplement: Supplementary file 2 [file pone.0000559.s002.doc]

Mice	CDDO-Im mg/Kg	total number of mice	deaths during treatment	lethality (%)
**wild-type**	5	8	0	0
	10	7	0	0
	20	3	1	33
**TRAF2DN/Bcl2**	5	8	0	0
**(Leukemic)**	10	9	4	44
	20	5	3	60
